# Congenital H-type Tracheo-oesophageal Fistula: An Institutional Review of a 10-year Period

**DOI:** 10.34763/jmotherandchild.20202404.d-20-00004

**Published:** 2021-07-13

**Authors:** Charu Tiwari, Nilesh Nagdeve, Rajendra Saoji, Nilesh Nama, Maaz Ahmed Khan

**Affiliations:** 1 Department of Paediatric Surgery, Government Medical College and Hospital, Nagpur, Maharashtra, India; 2 Department of General Surgery, Government Medical College and Hospital, Nagpur, Maharashtra, India

**Keywords:** congenital, fistula, H-type, trachea-oesophageal

## Abstract

**Background:**

Congenital H-type tracheo-oesophageal fistula (H-TOF ) accounts for 4%–5% of all congenital tracheo-oesophageal malformations. We present our experience in managing 18 cases with congenital H-TOF at a tertiary institute over a 10-year period.

**Methods:**

Records of all patients with congenital H-TOF managed from January 2009 to December 2018 in the Department of Paediatric Surgery at a tertiary institute were retrospectively analysed based on the age at presentation, gender, antenatal ultrasonography findings; birth history; details of previous hospitalisations, previous treatment details, presenting symptoms and associated anomalies; time to diagnosis; radiological investigations performed, bronchoscopy findings, intraoperative details, complications and postoperative follow-up.

**Results:**

Totally 18 patients with congenital H-TOF were managed over a 10-year period. There were 12 females and six males. Six patients had associated anomalies. There was wide variation in age at the start of symptoms (3 days–4 years) and presentation/referral to us (15 days–12 years). Four patients were diagnosed to have H-TOF at first admission. The most common presenting symptom was recurrent pneumonias (n=18). Bronchoscopy was done in all patients, and fistula was diagnosed and cannulated before surgery. The fistula was present at C8–T1 in 14 patients. The median age at surgery was 12 months. In 17 patients, the fistula was repaired by the cervical approach. There were two deaths, and 16 patients are doing well on median follow-up of 8 years.

**Conclusion:**

Congenital H-TOF should be considered in differential diagnosis while managing patients with recurrent lower respiratory tract infection and ‘coughing and choking episodes’; early diagnosis and management of the associated H-TOF is important for improved survival and outcome.

## Introduction

Tracheo-oesophageal fistula (TOF) without associated oesophageal atresia (EA), commonly called as H-type or N-type or isolated fistula, is a rare congenital anomaly and accounts for 4%–5% of all congenital tracheo-oesophageal malformations.^1^, ^2^, ^3^, ^4^ It was initially classified as Type 4 by Vogt in 1929^2^ and later reclassified as Type E by Gross in 1953.^3^ Because of its varied and nonspecific presentation, the diagnosis of H-TOF is difficult and often delayed, and in some cases, it may remain undiagnosed during childhood or, sometimes, even in adults.^5^, ^6^, ^7^ The usual presentation in infancy is choking during feeding, recurrent cough, cyanotic spells and/or unexplained abdominal distension.^1^ However, recurrent bouts of pneumonia, typically involving the right upper lobe, may also be the presenting manifestations.^1^ A high index of suspicion is important for early diagnosis. Oesophagography, computed tomography (CT) and bronchoscopy^8^, ^9^ are helpful in diagnosis. Surgical treatment involves division of the isolated TOF typically through a cervical approach. Given the rarity of this congenital oesophageal anomaly, only a few reports exist in the literature describing the long-term outcomes in these infants. Recently, two large multicentre reviews have been published, involving 14 centres over 10 years and four centres over 15 years, with a median of 9.5 and 5 patients per centre, respectively.^3^, ^4^

The aim of this study is to report our experience in terms of the diagnosis, management and postoperative outcomes in 18 cases of congenital H-TOF.

## Methods

This retrospective data analysis of patients of congenital H-type TOF was done in the Department of Paediatric Surgery at Government Medical College and Hospital, Nagpur, India. It included patients with an established diagnosis of congenital H-type TOF treated over a 10-year period, from January 2009 to December 2018. Diagnosis was established based on clinico-radiological findings and confirmed by bronchoscopic and surgical findings. Radiological diagnosis of H-type TOF was conducted by oesophagography or contrast-enhanced CT (CECT) on demonstration of the fistula between the trachea and the oesophagus . The findings were further confirmed during surgical exploration.

Data were obtained from the patients’ medical records, and the parameters analysed were age at presentation, gender, antenatal ultrasonography findings; birth history (birth weight, gestational age, symptoms at birth); details of previous hospitalisations, previous treatment details, presenting symptoms, associated anomalies; time to diagnosis, radiologic investigations performed, bronchoscopy findings, intraoperative details, complications and postoperative follow-up.

Descriptive analyses were performed. All data were analysed for normality; those with a normal distribution were reported as mean with standard deviation, and those with a skewed distribution were reported as median with range.

## Results

### Demographic characteristics

A total of 429 patients were managed for congenital oesophageal anomalies. Of these, 18 patients with H-type TOF were included in this study. Thus, the overall incidence of H-type TOF in patients with congenital oesophageal anomalies was 4.2%. The demographic details of these patients have been summarised in Table 1. There were six males and 12 females. The mean gestational age was 34 weeks (± 3 weeks), and the mean birth weight was 2,100 ± 300 g. Four of the patients were preterm babies. One neonate required bag-and-mask ventilation for respiratory distress just after birth. Classical frothing was absent in all patients, and there was no history suggestive of perinatal sepsis in any of the patients.

**Table 1:** Demographic characteristics of all 18 patients with H-TOF

Total patients with H-TOF	18 out anomalies of total oesophageal (4.2%)	
Age at presentation/referral (n=18)	<1 year	11
	1–3 years	4
	3–6 years	2
	6–12 years	0
	>12 years	1
Gender distribution (n=18)	Males	6
	Females	12
Number of previous hospitalisations (n=18)	1	8
	2	4
	3	5
	>3	1
Associated anomalies (n=6)	ASD	3
	VSD and PDA	1
	Renal agenesis	1
	Radial agenesis	1

H-TOF, H-type tracheo-oesophageal fistula; ASD, atrial septal defect; VSD, ventricular septal defect; PDA, patent ductus arteriosus.

Associated anomalies were seen in one third of the patients (n=6), including cardiac anomalies in four patients (atrial septal defect [ASD], ventricular septal defect [VSD] and patent ductus arteriosus [PDA]), unilateral renal agenesis and radial agenesis in one patient each.

### Presentation

The antenatal details were available only for six patients. Two antenatal sonograms had detected polyhydramnios. Twelve patients presented to the institute in the first year of life, and two-thirds of these patients were beyond the neonatal period. The youngest patient who had symptoms from the third day of life was referred for management on the 15th day, while the oldest patient was referred to us at 12 years of age and was symptomatic for 8 years. However, the age at presentation/ referral to us varied. Four patients were diagnosed to have H-type TOF at first admission. The diagnosis was missed in the remaining 14 patients, who required multiple admissions, i.e. one previous admission in four patients, two previous admissions in four patients and three previous admissions in five patients . Further, one patient had a history of five admissions prior to referral at our institute. Six patients had 7–15 days of previous hospital admissions. The mean duration spent in the hospital during previous admissions was 28 days (± 10 days). The reasons for the multiple hospitalisations have been summarised in Table 2.

**Table 2:** Overlapping reasons for previous hospitalisations in all patients with H-TOF

Presenting symptoms		No. of patients
**Respiratory symptoms**	Recurrent pneumonias	18
	Bouts of coughing and choking after feeds	16
	Cyanosis	8
**Failure to thrive**		14
**Abdominal symptoms**	Abdominal distension	6
	Constipation	8

H-TOF, H-type tracheo-oesophageal fistula.

All infants presented with a combination of coughing and choking and/or cyanosis during feeding. Patients had overlapping symptoms, such as recurrent pneumonias (n=18), bouts of coughing and choking after feeds (n=16) and failure to thrive (n=14). During previous hospitalisations, the patients received management for their respiratory infection; the total duration of antibiotics received was 18.6 ± 9.7 days; Two patients required ventilator support during previous hospitalisations due to recurrent lower respiratory tract infections. These patients were referred to us for bronchoscopy due to recurrent/persistent pneumonias (n=16), suspicious upper gastrointestinal (GI) findings (n=1) and other symptoms such as chronic cough and failure to gain weight (n=1).

**Figure 1:**
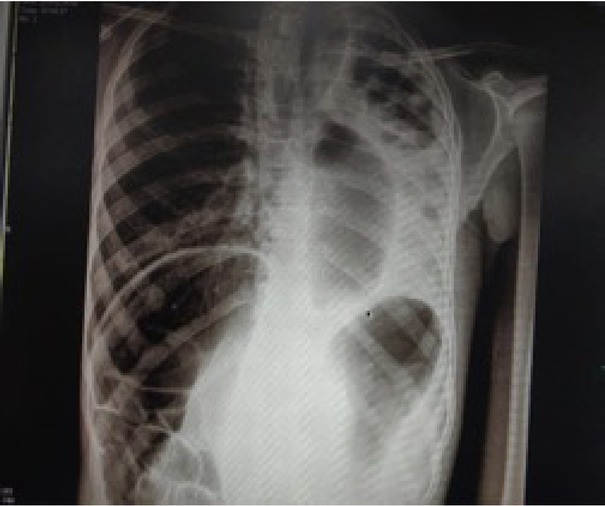
X-ray chest and abdomen of one patient showing complete collapse of the left lung, hyperinflation of the right lung and the Chilaiditi sign.

**Figure 2:**
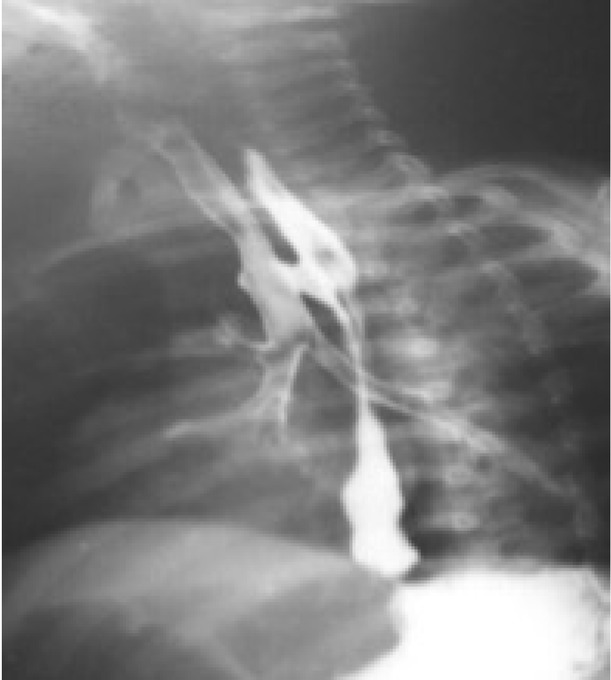
H-TOF identified in the upper gastrointestinal contrast study.

Eleven patients showed unilateral pneumonia on chest radiographs (patchy in eight patients and lobar in three patients). Three patients had bilateral lung involvement. Four patients who were beyond infancy had lung abscesses. One patient had complete collapse of the left lung and a hyperinflated right lung, with mediastinal shift towards the left side and the Chilaiditi sign in the abdomen (Fig 1). Abdominal X-rays showed dilated stomach and bowel loops in ten patients. Upper GI contrast study was done in all 18 patients; H-TOF was diagnosed in ten patients in this study (Fig 2). CECT of the thorax was done in four patients, and it revealed dilated oesophagus in all four patients, bilateral pneumonia in one patient, left lung abscess with hyperinflation of right lung in one patient, and H-TOF was diagnosed only in one patient. H-TOF, H-type tracheo-oesophageal fistula.

**Figure 3:**
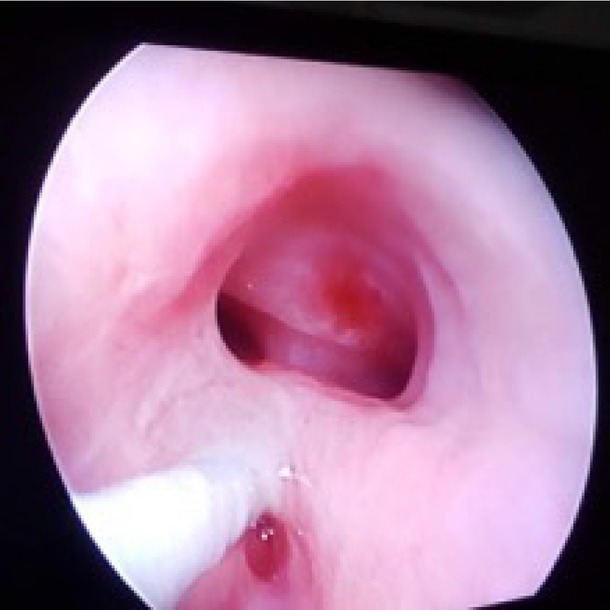
Bronchoscopy showing H-TOF at the C8–T1 level.

Bronchoscopy was done in all patients, and fistula was diagnosed and cannulated before surgery. The fistula was located at the C8–T1 level in 14 patients (Fig 3), at T1–T2 in three patients and just above the carina (T4 level) in one patient.

### Operative approach

**Figure 4:**
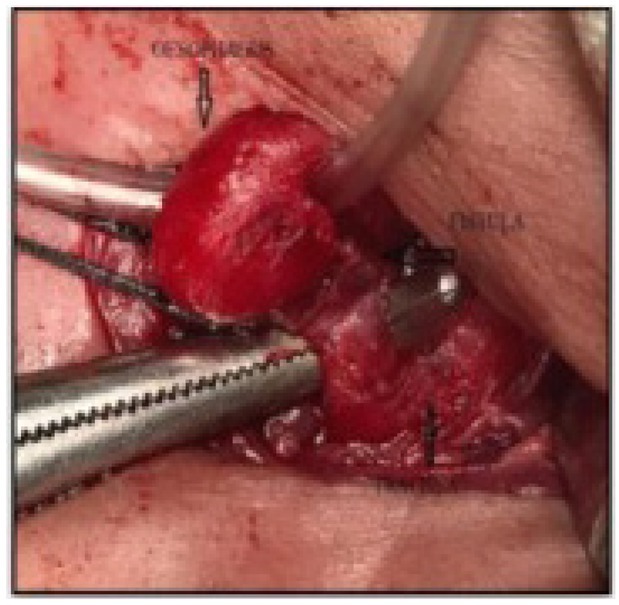
Intraoperative image showing the oesophagus and trachea, as well as identification of the fistula.

**Figure 5:**
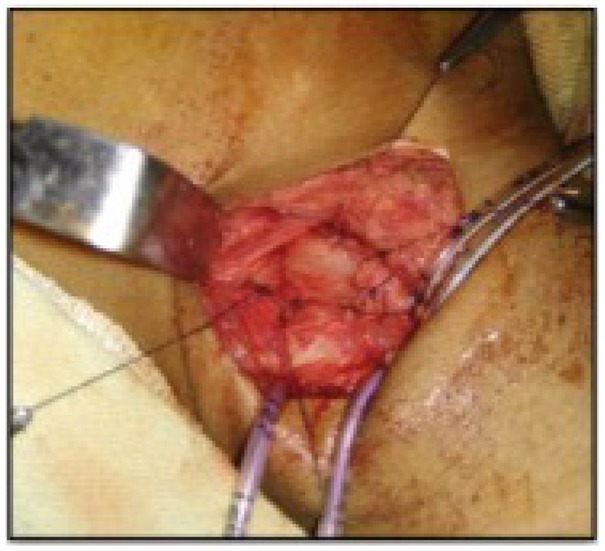
Intraoperative image showing the completed repair.

The median age at surgery was 12 months (range: 3 months–12 years). Seventeen patients underwent repair by the cervical approach (Fig 4). One patient required thoracotomy for repair. Bronchoscopy was done in all patients, and fistula was diagnosed and cannulated before surgery to assist in identification and manipulation of the fistula. The tracheal end was sutured with polypropylene, and the oesophageal end was sutured with absorbable polyglactin sutures (Fig 5). An interposition muscle graft from the sternocleidomastoid muscle was placed in all patients repaired through the cervical approach. The repair of the oesophagus was always done over an infant feeding tube/Ryles tube.

### Postoperative course

The postoperative course was uneventful in 12 patients; there were minimal complications in four patients. Two patients died in the early postoperative period; both had unilateral lobar consolidation preoperatively and were on preoperative ventilator support due to recurrent lower respiratory tract infections. They also had a history of requiring bag-and-mask ventilation in the postnatal period and had associated cardiac anomaly (ASD in one patient and VSD in the other). They developed acute respiratory failure in the early postoperative period.

The median postoperative and total hospital periods were 15 days (range: 8–30 days) and 20 days (range: 14–45 days), respectively. Nasogastric tube feeding was started in all patients from the fourth postoperative day. Postoperative upper GI contrast study was done on the seventh day. The median time to start oral feeds was 12 days (range: 6–15 days).

### Early postoperative complications

Four patients developed complications during hospital stay, which included minimal oesophageal leak (n=2), wound infection (n=1) and pneumonia (n=1). Oesophageal leak was managed conservatively with intravenous antibiotics and nasogastric tube feeding. Upper GI contrast study on the 15th day in all these patients showed healed repair. There was no anastomotic stricture in any of the patients on upper GI contrast study. Laryngoscopy was performed postoperatively in ten patients. Of these, one patient had unilateral paralysis, while the remaining patients had normal vocal cord function. Three patients had low-grade gastro-oesophageal reflux, which improved on conservative management. Oesophageal stenosis was noted in one patient. None of them had recurrence of fistula. All patients had weight gain, and there were no voice changes in any of them.

## Discussion

Although the symptoms of an H-type TOF are usually present from birth, the diagnosis of this condition is always a challenge. Absence of associated oesophageal atresia, configuration of the fistula and lack of awareness of this entity among the primary health service providers result in delay in the diagnosis. The condition is also not detected on antenatal ultrasonography. It may result in antecedent complications and multiple hospitalisations. In our study, only 22% patients were diagnosed in the newborn period and one-fifth were diagnosed during the first admission. Moreover, 55% patients required >2 hospitalisations. Various studies have indicated diagnosis rates of 43% in the first month, 70% during the first 6 months and between 83% and 90% during the first year of life.^4^,^10^ Some cases of H-type TOF may remain undiagnosed until late in infancy or childhood. Cases of H-type TOF are even described in adults.^9^, ^11^

The presentation is characterised by a clinical triad consisting of paroxysms of coughing and cyanosis precipitated by feeding, recurrent chest infection secondary to aspiration during feeding and abdominal distension due to gaseous distension of the gastrointestinal tract in those with a relatively large fistula. Majority of our patients presented with recurrent chest infection, choking and coughing during feeds. These symptoms though varying in severity and seemingly intermittent, are usually present from birth. The possibility of an H-type TOF should always be kept in mind by the physicians treating recurrent chest infections and/or choking and coughing during feeds. H-type TOF in older children and adults may result in other acquired diseases such as chronic obstructive pulmonary disease and bronchiectasis secondary to recurrent aspiration being the commonest.^7^ Older children in our study had bronchiectatic changes in the left lung at diagnosis. Moreover, the diagnosis of congenital H-type TOF is usually not included in the differential diagnosis of recurrent pneumonia and bronchiectasis in adults.^7^

The investigations in suspected case of H-type fistula are directed at not only demonstrating the fistula but also localising the site of the fistula, which is important to decide the surgical approach. Therefore, more than one modality of investigations may be required. Water-soluble contrast oesophagogram under fluoroscope is the first and most commonly used method to diagnose H-TOF. The close apposition of the trachea with the oesophagus, its small size, occlusion by oedema or food particles, presence of membrane hiding the orifice of the fistula and the obliquity of the H-type TOF with the oesophageal end at a lower level than the tracheal end may make the demonstration of fistula difficult.^8^, ^9^,^12^, ^13^ Several attempts and different positions of patients are often required for demonstration of fistula.^14^, ^15^

In our study, an oesophagogram was able to demonstrate the fistula in 55% of patients. The study may have the potential risk of aspiration pneumonia and should be performed with adequate emergency resuscitation at hand. High-resolution CT scan may be a useful adjuvant in those patients where contrast oesophagogram may not demonstrate fistula. Apart from demonstration of fistula, air-filled oesophagus was a constant finding on CT scan in our study. Though the CT scan was done in only four patients, it confirmed the diagnosis in all these patients. Magnetic resonance imaging (MRI) has also been used for the diagnosis and localisation of H-type TOF.^16^ Bronchoscopy is an important investigation that can be performed with fibre-optic endoscope or rigid ventilating bronchoscopes.^12^, ^17^ Preoperative bronchoscopy not only helps in identification of fistula but also in cannulation of the fistula, which helps in localising the fistula intraoperatively. Rigid bronchoscope was used in all patients in this study. We feel rigid bronchoscopy provides the advantages of better optics and control while cannulating the fistula . Nineteen (82.6%) of our patients had preoperative bronchoscopy, and a catheter (a Fogarty or ureteral catheter) was used to cannulate the fistula to aid its intraoperative localisation and dissection in 13 patients (56.5%). Use of the flexible bronchoscope has also been reported earlier for the operative localisation of fistula.^18^ The majority of isolated TOF can be managed through a cervical approach with moderate neck extension.^19^ Classically, H-type TOFs are located at the level of the thoracic inlet, and the oesophageal end of the fistula is usually located at a lower level than the tracheal end of the fistula. In our study, all repairs, except one, were achieved via a right-sided trans-cervical approach. Our study corroborates other studies reporting that the right-sided cervical approach is the preferred method to manage this condition.^16^, ^20^, ^21^ The cervical approach allows clear visualisation and protection of important structures such as the carotid artery, the internal jugular vein, the thyroid gland and the recurrent laryngeal nerve. Access through the right neck has the advantage of minimising damage to the right recurrent laryngeal nerve. Moreover, electrical nerve stimulation intraoperatively can be used to prevent damage to this nerve.^22^ One patient in our study, who had fistula just above the carina, required a right posterolateral thoracotomy approach. After repair, we interposed either a strap or the sternocleidomastoid muscle in all patients approached through the neck to prevent the overlapping of suture lines. Thoracoscopic repair of H-type TOF has been described in the literature as an alternative and less-invasive approach when compared with thoracotomy.^23^, ^24^, ^25^

This procedure is seldom performed as most H-type TOFs are located high and better approached through a cervical incision . Endoscopic closure of H-type TOF has been reported using various techniques such as tissue adhesives, electrocautery, sclerosants and laser.^26^, ^27^

However, surgical correction still remains the preferred modality in management. The postoperative results of our study are consistent with other reports as we found a relatively low rate of postoperative gastrointestinal complications 3, 5, 15

In our study oesophageal leak, gastro-oesophageal reflux and stenosis were observed in 11%, 16% and 5% patients, respectively. However, none of them required a second surgical procedure. No patient in our study developed recurrent TOF. It is difficult to say whether the use of interposition muscle flap reduced either leak or recurrence. It must be emphasised that the repair of an H-type TOF is not a simple procedure and should not be taken lightly as it is known to be associated with complications. One of the serious complications is damage to the recurrent laryngeal nerve. This must be borne in mind at the time of surgery and postoperatively as these patients may develop postoperative stridor . Eleven per cent of our patients had unilateral vocal cord paralysis on laryngoscopy, which fortunately recovered spontaneously. Most of the airway problems that occur after H-type TOF repair are due to vocal cord dysfunction caused by injury to the recurrent laryngeal nerve. It usually results in hoarseness and/or dysphagia but is often a transient phenomenon. A recently published multi-institutional review advocated early vocal cord evaluation for respiratory symptoms due to a higher incidence of airway problems after H-type fistula repair.^3^

The retrospective study design, small sample size and lack of long-term follow-up in some patients are some limitations of the present study. However, this study not only adds this rare condition to the current database but also substantiates the operative technique and postoperative results.

## Conclusion

Congenital H-type TOF must be considered in differential diagnosis when managing patients with recurrent lower respiratory tract infection and in patients with ‘coughing and choking episodes’. A high index of clinical suspicion is important for early diagnosis as delay in diagnosis is known to be associated with increased morbidity. Early surgical repair following contrast oesophagogram and bronchoscopy is the key to prevent morbidity.
